# Translation and validation of the Intermittent Catheterisation Difficulty Questionnaire (ICDQ) in an Arabic population

**DOI:** 10.1080/2090598X.2019.1694762

**Published:** 2019-12-03

**Authors:** Sameh Ghroubi, Jihène Chmak, Ons Borgi, Nedra El Fani, Soumaya El Arem, Mohamed Habib Elleuch

**Affiliations:** a Department of Physical Medicine and Rehabilitation, Hbib Bourguiba University Hospital, Sfax, Tunisia; b Unité de Recherche de L’évaluation des Pathologies de L’appareil Locomoteur UR12ES18, Université du Sud, Sfax, Tunisia

**Keywords:** Evaluation, Intermittent Catheterisation Difficulty Questionnaire, Arabic version, validation

## Abstract

**Objective**: To translate and validate linguistically an Arabic version of the Intermittent Catheterisation Difficulty Questionnaire (ICDQ) adapted to the Tunisian population.

**Patients and methods**: An Arabic translation and cultural adaptation of the ICDQ was obtained via the reverse translation method after two sets of comprehension tests within two groups of 10 patients. Psychometric validation included testing the questionnaire on a group of 30 patients. Intra-rater reliability was evaluated by the calculation of the intraclass correlation coefficient (ICC) for each item of the questionnaire. Cronbach’s α was used to assess internal consistency.

**Results**: The study included 30 patients (seven females, 23 males) with a mean (SD) age of 40.6 (15.3) years. The ICC was 0.96, demonstrating excellent intra-rater reliability. Cronbach’s α was 0.96 (>0.9) confirming an excellent correlation between the different items.

**Conclusion**: This work provides a translated, validated and Tunisian adapted version of the ICDQ that can be used to evaluate Tunisian patients’ difficulties with clean intermittent self-catheterisation in daily practice. We expect that this version will also be helpful for patients in other Arabic and North African countries, although such a hypothesis needs to be confirmed by further studies.

**Abbreviations:** CISC: clean intermittent self-catheterisation; ICDQ: Intermittent Catheterisation Difficulty Questionnaire; ASIA: American Spinal Injury Association; ICC: intraclass correlation coefficient

## Introduction

Clean intermittent self-catheterisation (CISC) is preferred for the management of neurogenic bladder [1,2]. Difficulties with this method are common and are the main reason for patients to interrupt self-catheterisation. The recognition of such difficulties and their objective evaluation are necessary steps to succeed in the choice or the adaptation of the catheter and to ensure a patient’s compliance to treatment. The Intermittent Catheterisation Difficulty Questionnaire (ICDQ) is a simple and validated questionnaire used for such evaluation [3]. The objective of the present study was to translate and validate linguistically an Arabic version of ICDQ that could be reliably used in daily practice with Tunisian patients.

## Patients and methods

### Study population

Patients who were referred between January and April 2016 to the neuro-urology outpatient clinic of our Physical Medicine and Rehabilitation Department for a neurogenic bladder were considered for inclusion in the study. The study inclusion criteria were: aged ≥18 years and using CISC for urinary retention resulting from a neurological disease. The exclusion criteria were: a confused mental state, bad compliance with CISC, and patients who lived far from the hospital or would have difficulties getting to it. For the included patients, the highest lesion was C6, knowing that patients with C6 injuries represent the highest international American Spinal Injury Association (ASIA) level able to manage CISC, with a success rate of 20–30% [4].

### The questionnaires

The ICDQ is a test developed and validated by a French team to evaluate catheter use, and patients’ difﬁculties during CISC; it includes 13 items. The response options are arranged on a 4-point Likert type scale, with zero indicating ‘none’ and 3 indicating ‘very difﬁcult’ in terms of use of the catheter. The frequency of the encountered difﬁculties is also estimated [3].

### Translation and cultural adaptation

From February 2017 to April 2017, we conducted the linguistic validation of the questionnaire, which was organised in several steps:

**Step 1**: Establish the initial Arabic translation of the questionnaire from the French version using the reverse translation method (forward translation–backward translation).

Direct translation was entrusted to two bilingual professional translators working as a team. These two translators have as their mother tongue Arabic, which is the target language of the translation. They were given the instruction to emphasise meaning rather than literal translation.

The translators were asked to prepare a written report including the translation, as well as comments on their work, choices, difficulties encountered, and uncertainties. The version thus obtained V0 was criticised and evaluated by a committee of experts comprised of six physicians and a bilingual volunteer patient representative of the target population. The questionnaire’s administering method was chosen by the committee of experts to be adapted to the intellectual level of the target population (operational equivalence).

At the end of this stage, the committee of experts validated version V1 of the questionnaire intended to be tested with a first group of patients.

**Step 2**: Perform a first set of comprehension tests in a group of 10 patients.

Version V1 was administered as a hetero-questionnaire to 10 volunteer patients by a physician to verify the acceptability and comprehension of the items. Items were spelled out word for word as they were written. The investigator could repeat the questions but could not change the words. Patients included in this test should answer two yes/no questions for each item: ‘Was the item easy to understand?’ and ‘Is the item acceptable in terms of discretion?’. In case of difficulty, patients were asked to identify the difficult words and to offer suggestions of reformulation.

**Step 3**: Prepare a revised version of the questionnaire

After the first set of comprehension tests and based on the patients’ reformulation suggestions, the committee of experts validated the second version of the questionnaire V2, destined to be tested within a second group of patients.

**Step 4**: Perform a second set of comprehension tests in a group of 10 other patients.

The 10 patients in this second set of comprehension tests were all voluntary and different from those tested in the first series, and meeting the inclusion and exclusion criteria. The ability to perform CISC was verified by the paper-pencil test [5].

The acceptability of the questionnaire, as well as the understanding of the items, was evaluated again according to the procedure described above.

**Step 5**: Prepare the final version of the questionnaire

Based on the data provided by the second set of comprehension tests, the committee of experts was able to prepare a final version in Arabic, which was to be submitted to a backward-translation in French.

**Step 6**: Conduct a backward-translation of the final version.

The final version V3 of the questionnaire was entrusted to a third bilingual professional translator to translate it from Arabic into French.

**Step 7**: Validation of the final version of the questionnaire by the committee of experts.

To validate the final version of the questionnaire, the backward-translation was compared to the original version by the committee of experts.

### Psychometric validation

Between April and June 2017, the final version of ICDQ was tested in a prospective study including 30 voluntary patients, different from those tested in the first series, meeting the inclusion and exclusion criteria. All patients were able to perform CISC with a paper-pencil test superior to 10/15. Demographic and clinical data were collected for each patient including: gender, age, literacy, disease, period during which CISC had been used, type of catheter used, and number of CISCs per day.

The questionnaire was administrated twice to the 30 patients by the same investigator at an interval of 7–10 days (test–retest). This period was judged to be sufficiently long for the patients to forget the questionnaire and sufficiently short to avoid any change in their clinical status. A questionnaire is considered reliable if a certain degree of agreement and similarity is established between the two measurements. This intra-rater reliability (repeatability, concordance) was evaluated by the calculation of the intraclass correlation coefficient (ICC) for each item. When the ICC is <0.5 this indicates poor reliability and the item is eliminated [6].

The comprehension via the number of missing data and acceptability in terms of discretion and time consumption were evaluated.

Cronbach’s α was used to assess internal consistency, which means how well the test measures what it should [7]. Internal consistency is judged to be acceptable when the Cronbach’s α is ≥0.7 [7].

## Results

### Translation and cultural adaptation

The correspondence between words is not always possible given the vocabulary and the grammar appropriate to each language. In this context, when a term is untranslatable, we replaced it by a group of words of equivalent meaning.

Difficult words in Arabic were formulated in the Tunisian dialect and were put in parentheses for explanatory purposes.

After the first cognitive debriefing, three items were modified and no item was eliminated. Comprehension and acceptance of the questionnaire were good at the second cognitive debriefing, thus, no item was modified.

### Population

The patients’ clinical and demographic characteristics are summarised in Table 1. In total, 30 patients (seven females, 23 males) were enrolled in this study. Their mean (SD) age was 40.6 (15.3) years. All patients had a neurological disease (multiple sclerosis, spinal cord injury, cauda equina syndrome, spina biﬁda, myelitis, neuromeningeal tuberculosis, and neuromeningeal spondylodiscitis).

**Table 1.** Patients’ clinical and demographic characteristics.

Characteristic	Number of patients	Number of women/men	Age, years, mean (SD)	ICDQ score at the first measurement, median (IQR)	Duration of CISC, months, median (IQR)
Multiple sclerosis	5	2/3	53.6 (17.7)	4 (1.5–9.5)	100 (13.5–174)
Spinal cord injury	18	0/18	41 (13)	3(1–8.25)	44.5 (12–59)
Cauda equina syndrome	1	1/0	-	-	-
Spina biﬁda	2	2/0	17 (5.6)	9 (7–10)	
Myelitis	2	0/2	27 (5.6)	7.5 (5–9)	47.5 (41–100)
Neuromeningeal tuberculosis	1	1/0	-	-	
Neuromeningeal spondylodiscitis	1	1/0	-	-	

Four patients were illiterate, 14 patients had elementary school level, and eight had high school level. Only four were college graduates.

All patients used a hydrophilic-coated catheter. Thirteen patients resorted to both spontaneous micturition and CISC, and 17 patients could not normally urinate and used only CISC.

The mean (SD) number of CISCs per a day was 4.9 (2.1). The patients had been using CISC for a mean (SD) period of 85.5 (75.8) months.

### Psychometric validation

The comprehension of the questionnaire was good. There were missing data. The acceptability in terms of discretion and time consumption was excellent (100%).

The mean ICDQ score at the first and second measurement (7–10 days later) was 4 with an interquartile range (IQR) of 1.75–9.25 and 1–8.25, respectively. The ICC was 0.96, demonstrating excellent intra-rater reliability (Table 2) [6].

**Table 2.** ICC for each item of the ICDQ questionnaire.

Items	ICC
Item 1	0.97
Item 2	0.98
Item 3	0.95
Item 4	0.98
Item 5	0.97
Item 6	1
Item 7	0.91
Item 8	0.92
Item 9	0.98
Item 10	0.98
Item 11	0.9
Item 12	1
Item 13	0.98

Cronbach’s α was 0.96 (>0.9), confirming an excellent correlation between the different items [8].

## Discussion

**Figure 1.**
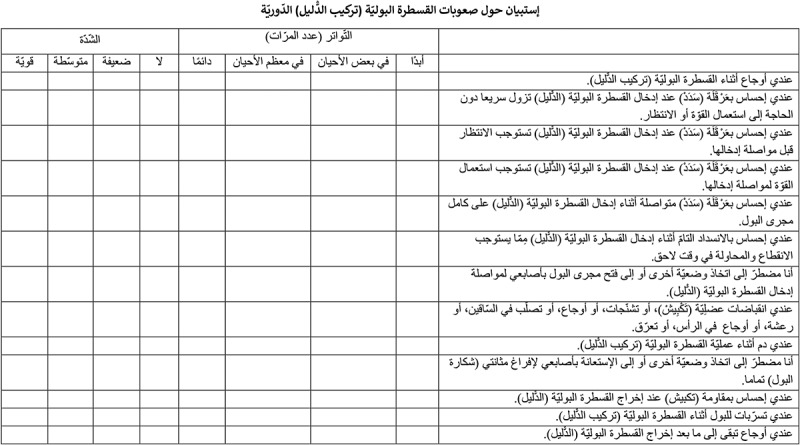
Final Arabic (Tunisian) version of the ICDQ.

This work provides a translated and Tunisian adapted Arabic version of the ICDQ (Figure 1). The translation process was organised in two steps: the actual translation of the items and the adaptation of the questionnaire to the cultural context and features of the target population. Given that there is no consensus in the translation methodology [9,10], we chose to adopt the forward/backward translation method. We used easy literary Arabic terms because literary Arabic is the official language that unifies many countries. The Tunisian dialect was used in parentheses to explain and simplify the technical and difficult words and to make the questionnaire culturally well fitted to the Tunisian population where the rate of illiteracy is in double figures in people aged >30 years and >40% in those aged >60 years [11]. This method has been used in several studies [10,12] and recommended at this stage of translation [13]. The pre-test study allowed us to detect the terms that might be poorly or not at all assimilated by patients, in particular the illiterate. It is of essential importance. It often leads to new adaptations, and allows for an initial assessment of the scales. Several preliminary studies are sometimes needed, in order to produce usable questionnaires [14]. The inclusion of a volunteer patient helped to identify comprehension difficulties and the potential sources of confusion within the items. The cultural adaptation of the questionnaire changed neither the number of items nor their pattern.

As to the metrological qualities of the questionnaire, we studied the intra-rater repeatability using the ICC. The ICC was 0.96, with the intra-rater concordance judged as excellent. An ICC of >0.5 is considered sufficient and suggested satisfactory repeatability of the Arabic-language version of the questionnaire [6].

The inter-rater concordance (repeatability) was not evaluated. Such evaluation was found unnecessary because, although ICDQ was hetero-administrated, the investigator was given the instruction to only read the items as they were written and explained in both literary and dialectical Arabic. Thus, the investigator did not need to interfere for explanation.

The internal consistency study, through Cronbach’s α, demonstrated excellent internal consistency (α = 0.96, >0.9). In the original French version of the ICDQ the Cronbach’s α was 0.94 [3].

## Conclusions

The ICDQ is a simple and validated questionnaire for the evaluation of patients’ difficulties with self-catheterisation. Our present study resulted in a linguistically validated Arabic version of ICDQ, which is adapted to the Tunisian population. This Arabic version has excellent internal consistency and its intra-rater concordance is satisfactory. Its use in daily practice with Tunisian patients will help to identify specific difficulties with CISC to better adapt the treatment or propose specific guidance or advice about CISC. We are expecting that this version will also be helpful for patients in other Arabic and North African countries, although such a hypothesis needs to be confirmed by further studies.

## References

[1] Groen J, Pannek J, Castro Diaz D, et al. Summary of European Association of Urology (EAU) guidelines on neuro-urology. Eur Urol. 2016;69:324–333. 26304502 10.1016/j.eururo.2015.07.071

[2] Lane GI, Elliott SP. Safely avoiding surgery in adult neurogenic bladder. Curr Bladder Dysfunct Rep. 2018;13:16–1779.

[3] Guinet-Lacoste A, Jousse M, Tan E, et al. Intermittent catheterization difficulty questionnaire (ICDQ): A new tool for the evaluation of patient difficulties with clean intermittent self-catheterization. Neurourol Urodyn. 2016;35:85–89. 25327888 10.1002/nau.22686

[4] Asayama K, Kihara K, Shidoh T, et al. The functional limitations of tetraplegic hands for intermittent clean self-catheterisation. Paraplegia. 1995;33:30–33. 7715950 10.1038/sc.1995.7

[5] Amarenco G, Guinet A, Jousse M, et al. Pencil and paper test: a new tool to predict the ability of neurological patients to practice clean intermittent self-catheterization. J Urol. 2011;185:578–582. 21168886 10.1016/j.juro.2010.09.106

[6] Koo TK, Li MY. Guideline of selecting and reporting intraclass correlation coefficients for reliability research. J Chiropr Med. 2016;15:155–163. 27330520 10.1016/j.jcm.2016.02.012 PMC4913118

[7] Cronbach LJ. Coefficient alpha and the internal structure of tests. Psychometrika. 1951;16:297–334.

[8] Tavakol M, Dennick R. Making sense of Cronbach’s alpha. Int J Med Educ. 2011;2:53–55. 28029643 10.5116/ijme.4dfb.8dfd PMC4205511

[9] Guillemin F, Bombardier C, Beaton D. Cross-cultural adaptation of health-related quality of life measures: literature review and proposed guidelines. J Clin Epidemiol. 1993;46:1417–1432. 8263569 10.1016/0895-4356(93)90142-n

[10] Offenbaecher M, Ewert T, Sangha O, et al. Validation of a German version of the disabilities of arm, shoulder, and hand questionnaire(DASH-G). J Rheumatol. 2002;22:401–402. 11838867

[11] Institut National de la Statistique. Recensement général de la population et de l’habitat 2014, Volume 4: caractéristiques d’education de la population. [cited 2019 10]. Available from: http://www.ins.tn/sites/default/files/publication/pdf/RGPH-national-education-site.pdf

[12] Yahia A, Guermazi M, Khmekhem M, et al. Translation into Arabic and validation of the ASES index in assessment of shoulder disabilities. Ann Phys Rehabil Med. 2011;54:59–72. 21354384 10.1016/j.rehab.2010.12.002

[13] Guermazi M. Le concept d’incapacité fonctionnelle adaptation d’outils de mesure à la langue et au mode de vie. Thèse de sciences, Bourgogne; 2004.

[14] Bullinger M, Alonso J, Apolone G, et al. Translating health status questionnaire and evaluating their quality: the IQOLA project approach. J Clin Epidemiol. 1998;51:913–923. 9817108 10.1016/s0895-4356(98)00082-1

